# Exploring Urinary Extracellular Vesicles and Immune Mediators as Biomarkers of Kidney Injury in COVID-19 Hospitalized Patients

**DOI:** 10.3390/diagnostics12112600

**Published:** 2022-10-27

**Authors:** Thalia Medeiros, Lilian Santos Alves, Mauro Jorge Cabral-Castro, Alice Ramos Oliveira Silva, Analúcia Rampazzo Xavier, Dylan Burger, Jorge Reis Almeida, Andrea Alice Silva

**Affiliations:** 1 Multiuser Laboratory for Research Support in Nephrology and Medical Sciences (LAMAP), Faculty of Medicine, Universidade Federal Fluminense, Niterói 24033-900, Rio de Janeiro, Brazil; 2 Department of Pathology, Faculty of Medicine, Universidade Federal Fluminense, Niterói 24033-900, Rio de Janeiro, Brazil; 3 Instituto de Microbiologia Paulo de Góes, Universidade Federal do Rio de Janeiro, Rio de Janeiro 21941-901, Rio de Janeiro, Brazil; 4 Kidney Research Centre, Department of Cellular and Molecular Medicine, The Ottawa Hospital Research Institute, University of Ottawa, Ottawa, ON K1H 8M5, Canada; 5 Department of Clinical Medicine; Faculty of Medicine, Universidade Federal Fluminense, Niterói 24033-900, Rio de Janeiro, Brazil

**Keywords:** COVID-19, hemodialysis, urine, extracellular vesicles, cytokines

## Abstract

Kidney injury is an important outcome associated with COVID-19 severity. In this regard, alterations in urinary extracellular vesicles (uEVs) could be detected in the early phases of renal injury and may be reflective of the inflammatory process. This is an observational study performed with a case series of COVID-19 hospitalized patients presenting mild-to-critical disease. Total and podocyte-derived uEVs were identified by nanoscale flow cytometry, and urinary immune mediators were assessed by a multiplex assay. We studied 36 patients, where 24 (66.7%) were considered as mild/moderate and 12 (33.3%) as severe/critical. Increased levels of total uEVs were observed (*p* = 0.0001). Importantly, total uEVs were significantly higher in severe/critical patients who underwent hemodialysis (*p* = 0.03) and were able to predict this clinical outcome (AUC 0.93, *p* = 0.02). Severe/critical patients also presented elevated urinary levels (*p* < 0.05) of IL-1β, IL-4, IL-6, IL-7, IL-16, IL-17A, LIF, CCL-2, CCL-3, CCL-11, CXCL-10, FGFb, M-CSF, and CTAcK. Lastly, we observed that total uEVs were associated with urinary immune mediators. In conclusion, our results show that early alterations in urinary EVs could identify patients at higher risk of developing renal dysfunction in COVID-19. This could also be relevant in different scenarios of systemic and/or infectious disease.

## 1. Introduction

Kidney injury can be observed in up to 40% of hospitalized patients with coronavirus disease-19 (COVID-19), where about 20% of them require renal replacement therapy [1,2]. In this context, several studies have demonstrated that acute kidney injury (AKI) associated with COVID-19 can lead to higher mortality rates, especially in severe cases and in association with older age, male gender, and the presence of comorbidities [3,4]. Several factors are involved in the renal pathophysiology of SARS-CoV-2 infection. These include cytopathic effects through viral entry using the angiotensin-converting enzyme (ACE) receptors in podocytes, proximal tubule epithelial cells, and collecting ducts [5]. The unbalance of the renin-angiotensin-aldosterone system can also occur, with the activation of coagulation pathways and damage to the renal vasculature [6]. Furthermore, the kidney can also be affected by the proinflammatory and prothrombotic state promoted by SARS-CoV-2 infection [7,8].

Extracellular vesicles (EVs) are nanosized biologically active cell fragments released to the extracellular milieu under stress conditions, which may reflect an early phase of cellular injury [9,10]. Urinary EVs (uEVs) are considered promising prognostic biomarkers of renal dysfunction [11,12], and previous studies have demonstrated that podocyte- and tubule-specific uEVs can be significantly altered in different etiologies of kidney disease [13,14,15]. Nevertheless, little is known about the dynamics of uEV release in viral nephropathies, including SARS-CoV-2 infection, where uEVs could be important early indicators of renal dysfunction.

Previous studies have already demonstrated the importance of evaluating immune mediators in urine samples of patients with renal disease. In this regard, increased urinary excretion of cytokines such as interleukin (IL)-6, tumor necrosis factor-α (TNF-α), monocyte chemoattractant protein-1 (MCP-1), interferon-γ 10-induced protein (IP-10), and IL-10 were reported in patients with sepsis [16], acute interstitial nephritis [17], diabetic nephropathy [18], and systemic lupus erythematosus [19]. In COVID-19, despite the cytokine storm being widely studied as a pathophysiological mechanism associated with disease severity [20], few studies have evaluated the urinary concentrations of these mediators in association with potential kidney damage.

In this study, our aim was to assess the levels of uEVs and urinary immune mediators (cytokines, chemokines, and growth factors) in hospitalized COVID-19 patients in parallel with classical laboratory parameters. Moreover, we investigated possible associations between these biomarkers and COVID-19 severity, including the need for hemodialysis as a clinical outcome. Lastly, we investigated possible associations between uEV release and urinary immune mediators.

## 2. Materials and Methods

### 2.1. Study Design and Data Collection

We performed a pilot study which included adult patients diagnosed with COVID-19 admitted at the Hospital Universitario Antonio Pedro (HUAP- Niteroi, Rio de Janeiro, Brazil) from May to September 2020. Exclusion criteria was unavailability of urine samples and patients with previous diagnosis of stage 4/5 chronic kidney disease (CKD) or prostate cancer.

Demographic, clinical, and laboratory data were obtained by analyzing patients’ medical records. For further analysis, we stratified patients into groups according to the clinical presentation upon admission based on the National Institutes of Health (NIH) classification: patients with mild/moderate disease (fever, cough, sore throat, headache, muscle pain, nausea, vomiting, diarrhea, loss of taste and smell, oxygen saturation ≥ 94%, and no changes in imaging tests) and severe/critical disease (oxygen saturation < 94% in ambient air, >50% of pulmonary infiltrates, invasive mechanical ventilation, intensive care unit admission with respiratory failure, septic shock, thrombotic disease, and/or multiple organ dysfunction) [21].

For the healthy control (HC) group, we used urine from healthy volunteers with no history of arterial hypertension, diabetes, kidney disease and recent infection at the time of sample collection.

### 2.2. Sample Collection and Routine Laboratory Tests

Urine samples were obtained from the Clinical Pathology Service (SPC-HUAP) during the first week of hospitalization after performing routine tests required by the medical team. After an initial centrifugation (2500× *g* for 10 min at room temperature), urine supernatant was obtained and stored at −80 °C. Samples were only thawed once, at the moment of analysis.

Urinary creatinine, total protein, and albumin were measured in all samples using the Creatinina cinética AA, ProtiU/LCR, and Microalbuminuria commercial kits (Wiener Laboratorios SAIC, Santa Fe, Argentina), respectively. All tests were performed according to the manufacturer’s instructions.

### 2.3. Detection of uEVs by Nanoscale Flow Cytometry

For the detection of total and podocyte-derived uEVs, each urine sample was initially thawed at room temperature and centrifuged at 20,000× *g* for 20 min at 4 °C. The pellet was resuspended in Annexin V Binding buffer (Invitrogen, cat. V13246) with Anti-Annexin-V FITC (1:50, Biolegend, San Diego, CA, USA) and Anti-human Podoplanin APC (1:100, eBioscience, San Diego, CA, USA). After a second centrifugation and resuspension, samples were acquired using the CytoFLEX S flow cytometer (Beckman Coulter, Brea, CA, USA). The identification of uEVs was based on size, using calibration beads (100–900 nm, MEGAMIX-Plus FSC, Biocytex, Marseille, Provence, France), and antibody positivity. Further analysis was performed using the software FlowJo version 9/10 (FlowJo LLC, Becton Dickinson, Franklin Lakes, NJ, USA). Importantly, uEV levels were normalized to urinary creatinine of all samples for the correction for any differences in urine volume [22].

### 2.4. Assessment of Urinary Immune Mediators

Urinary levels of immunological mediators were assessed using the high-performance Bio-Plex Pro Human Cytokine Screening Panel 48 Plex kit (Bio-Rad, Hercules, CA, USA). We analyzed urinary levels of (i) chemokines [C-C chemokine ligand (CCL)-2/monocyte chemoattractant protein 1/MCP-1, CCL-3/MIP-1 α macrophage inflammatory protein 1α, CCL-4/MIF-1β macrophage inflammatory protein 1β, CCL-5/RANTES, CCL-7/MCP-3, CCL-11/eotaxin, CCL-27/CTACK cutaneous T cell-attracting chemokine, C-X-C ligand 1 (CXCL1/GRO-α), C-X-C interferon chemokine ligand (CXCL)-8 (IL-8), -9 (MIG), and CXCL-10/IP-10 gamma-induced protein 10, CXCL-12/SDF-1α stromal cell-derived factor 1]; (ii) cytokines [tumor necrosis factor-α/TNF-α, interferon α/IFN-α2, interferon γ/IFN-γ, interleukin 1/IL-1Ra receptor antagonist, interleukin 2/IL-2Rα receptor antagonist; migration inhibitory factor/MIF, leukemia inhibitory factor/LIF, and interleukins (IL)-1α, -1β, -2, -3, -4, -5, -6, -7, -9, -10, -12p40, -12p70, -13, -15, -16, -17A, and -18]; and (iii) growth factors [hepatocyte growth factor/HGF, granulocyte-macrophage colony stimulating factor/GM-CSF; granulocyte colony stimulating factor/G-CSF; basic fibroblast growth factor/FGFb; platelet-derived growth factor BB/PDGF-BB; nerve growth factor-beta/β-NGF, stem cell factor/SCF, and stem cell growth factor-beta/SCGF-β]. Reading was performed using the MAGPIX Luminex instrument (Luminex, Austin, TX, USA), and results were expressed in pg/mL.

### 2.5. Statistical Analysis

Data were expressed as mean ± SD or n (%). Differences between two independent groups were assessed by T-student or one-tailed Mann–Whitney tests according to the variable’s distribution. For more than two groups, we used the Kruskal–Wallis test with Dunn’s post-test. Differences between frequencies were analyzed with Chi-square test.

We performed the receiver operating characteristic curve (ROC) for urinary measurements in order to investigate their ability to predict clinical outcomes. The cut-offs were determined from the ROC analysis according to Youden’s index, which maximized both sensitivity and specificity [maximum = (sensitivity + specificity) − 1] [23]. Moreover, we designed a heatmap to demonstrate the landscape of urinary immune mediators. For this, we calculated the z-scores by subtracting the observed value of the selected cytokine by the mean and dividing by the standard deviation [(Z = (X − μ)/σ, where X is the observation, μ is the mean, and σ is the SD]. Then, we used the z-score to plot the heatmap [24].

To investigate potential confounding factors on urinary parameters, we performed a simple linear regression using total uEVs as the dependent variable and age/diabetes/hypertension as the predictor variables [25]. Lastly, correlations were performed to investigate possible associations between variables. *p*-values were considered significant when <0.05. Statistical analyses were conducted using the Prism software (GraphPad Software 8.0, San Diego, CA, USA) and R.3.2.2 (R Core Team).

## 3. Results

From April to August 2020, we received respiratory tract samples of 381 hospitalized patients to elucidate COVID-19 cases. Of the 126 positive patients, we were able to obtain urine samples from 41 patients. A total of 5 patients were excluded due to history of prostate cancer (n = 4) or stage 4 CKD (n = 1). Thus, for the analysis of uEVs and immune mediators, we included 36 patients.

Overall, we observed a mean age (±SD) of 56.3 ± 18.5 years and 20 patients were female (55.6%). According to the NIH criteria for COVID-19 severity, we identified that 24 (66.7%) patients had a mild/moderate clinical presentation and 12 (33.3%) presented a severe/critical disease at hospital admission. Demographic, laboratory, and clinical characteristics are presented in Table 1.

As expected, we observed that patients with severe/critical COVID-19 were more frequently diabetic (*p* = 0.04), in addition to requiring intensive care (*p* = 0.01) and hemodialysis more often (*p* = 0.02). Routine laboratory assessment at admission showed that these patients presented significantly higher levels of urea (*p* = 0.03), proteinuria (uPtn/uCr; *p* = 0.001), and albuminuria-to-creatinine ratio (ACR; *p* < 0.001). Lastly, the frequency of death by COVID-19 was also higher in the same group (*p* = 0.004).

**Figure 1:**
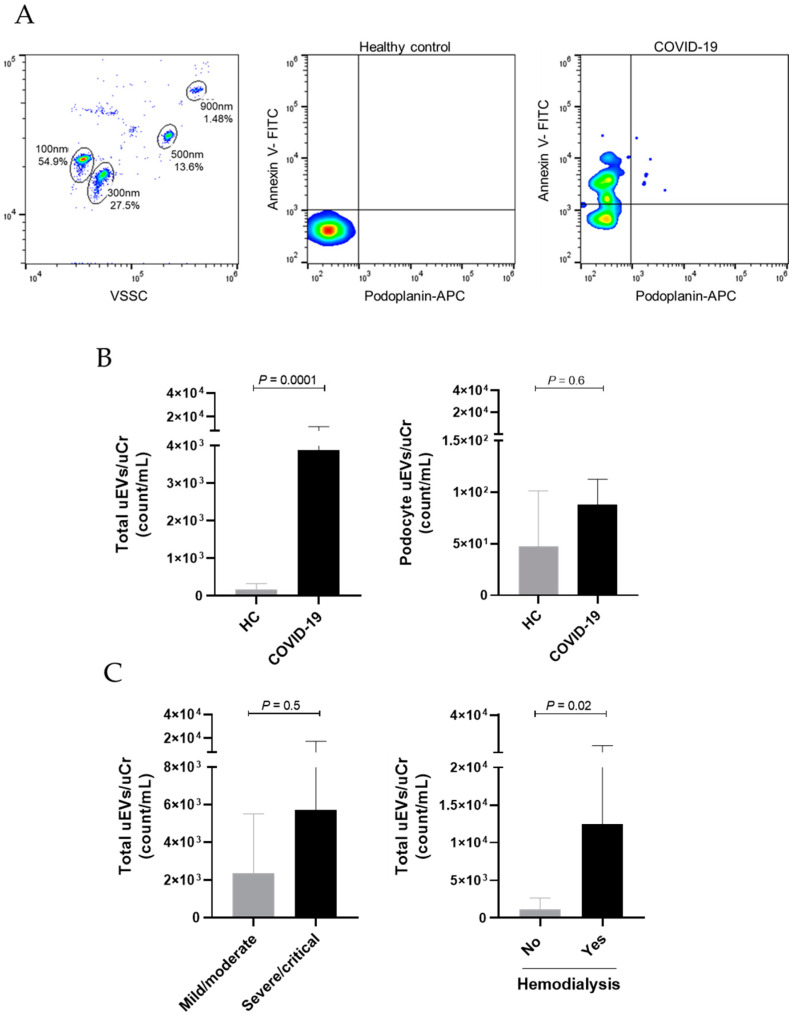
Urinary total and podocyte-derived EVs in COVID-19 hospitalized patients and healthy controls. (**A**) Flow cytometry gating strategy shows EV identification based on calibration with size beads (100 nm–900 nm) and representative dot plots demonstrate the characterization of total uEVs (Annexin V^+^) and podocyte-derived uEVs (Annexin V^+^ Podoplanin^+^) in a healthy control (HC) and in a patient with COVID-19. (**B**) Comparison of uEVs between HC and COVID-19 patients. (**C**) Comparison of total uEVs according to COVID-19 severity and need for hemodialysis. Differences between groups were assessed using Mann–Whitney test.

In regards to uEV analysis, we identified that COVID-19 patients presented significantly higher levels of total uEVs when compared to healthy controls (*p* = 0.001). Podocyte-uEV counts were also higher in the COVID-19 group, but significant differences were not observed (47.4 ± 53.9 vs. 74.4 ± 130.2 count/mL, *p* = 0.6). When evaluating total uEVs according to COVID-19 severity, we observed that severe/critical patients presented higher counts (2359 ± 3157 vs. 5717 ± 11.648 count/mL, *p* = 0.5), but with no statistical significance. Importantly, we also identified that patients who needed hemodialysis during disease progression presented significantly elevated levels of total uEVs at hospital admission when compared to the other severe/critical COVID-19 patients (*p* = 0.02). Figure 1 shows the comparison of uEV levels between groups.

**Figure 2:**
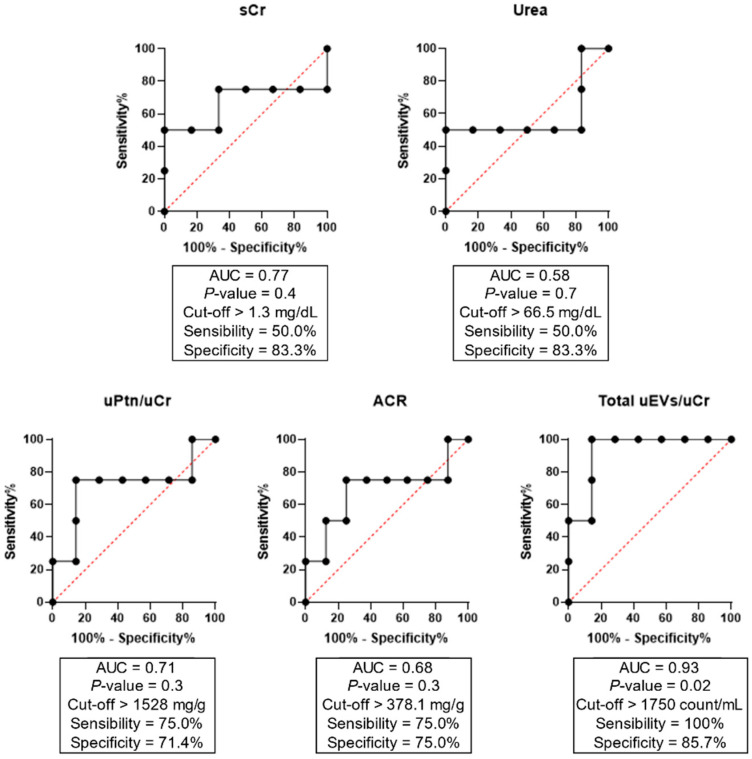
ROC curves of total uEVs, proteinuria, and albuminuria for the prediction of hemodialysis as a clinical outcome in COVID-19 severe/critical patients. ACR = albumin-to-creatinine ratio; AUC = area under the curve; Cr = creatinine; Ptn = protein; u = urinary.

Of note, a sensitivity analysis for the potential influence of age and comorbidities such as diabetes and hypertension on uEV excretion was conducted. We observed no effect of age (*p* = 0.73), diabetes (*p* = 0.88), and hypertension (*p* = 0.56) on total uEV levels (Table S1). Our next step was to assess the potential of uEVs, as well as other laboratory renal function parameters, in predicting clinical outcomes when evaluated early at hospital admission (Figure 2). ROC curves showed that only total uEVs presented a predictive power for hemodialysis, with AUC values of 0.93, sensibility of 100%, specificity of 95.7%, and *p* = 0.02. On other hand, serum Cr, urea, uPtn/uCr ratio, and ACR did not show significant results.

**Figure 3:**
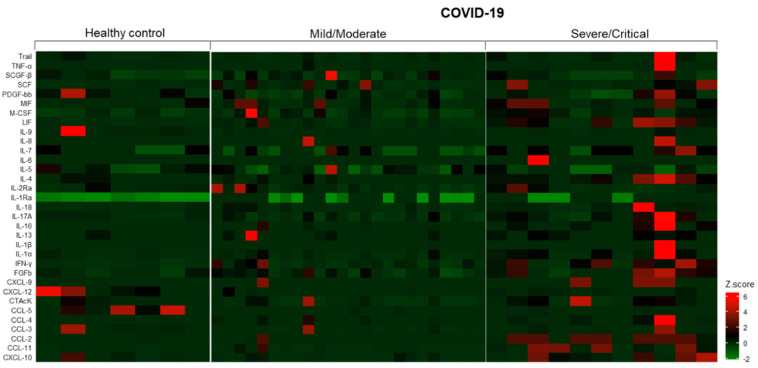
Landscape of urinary immune mediators in healthy controls and COVID-19 hospitalized patients. The z-score for each mediator was calculated by subtracting the observed value by the mean and dividing by the standard deviation.

In regards to the analysis of urinary immune mediators, of the 48 parameters included in the multiplex assay, we observed that the levels of 14 mediators (IL-2, IL-3, IL-10, IL-12p40, IL-12p70, IL-15, TNF-β, CCL-7, IFN-α2, G-CSF, GM-CSF, VEGF, β-NGF, and GRO-α) were under the lower limit of detection. Thus, for further analysis, we included 34 immune mediators. Overall, as shown in Figure 3, we identified that COVID-19 patients with severe/critical disease presented significantly higher urinary levels of IL-1β, IL-4, IL-6, IL-7, IL-16, IL-17A, LIF, CCL-2, CCL-3, CCL-11, CXCL-10, FGFb, M-CSF, and CTAcK when compared to HC and/or COVID-19 patients with mild/moderate disease. In contrast, the levels of IL-9, CCL-5, and CXCL-12 were significantly reduced in the same group. *p*-values are described in Table S2. It is important to mention that differences were not observed when we evaluated patients who underwent hemodialysis in comparison with those who did not require renal replacement therapy.

Lastly, we identified that urinary levels of immune mediators were significantly associated with total uEV count. Strong correlations (r ≥ 0.8) were observed between total uEVs and proinflammatory cytokines such as TNF-α, IL-1α, IL-1β, IL-16, and IL-17A and chemokines and other biomarkers such as CXCL-2, TRAIL, and HGF. These results are demonstrated in Table 2.

## 4. Discussion

It is known that severe COVID-19 is a systemic disease. Specifically on the kidneys, SARS-CoV-2 can promote direct effects, but other factors can also lead to AKI in COVID-19 patients, such as ischemia, systemic inflammation, and endothelial dysfunction [5,7]. Thus, since uEVs have been studied as early biomarkers of kidney injury, we aimed to investigate uEV levels in a cohort of Brazilian COVID-19 patients admitted at a university hospital during the first phase of the pandemic, according to clinical outcomes and in association with urinary inflammatory measures.

Our results showed that COVID-19 patients presented significantly higher levels of total uEVs in samples collected during the first days of hospitalization. This was accompanied by elevated serum urea, proteinuria, and albuminuria, especially in patients with severe/critical disease. In this context, urinary abnormalities such as proteinuria, hematuria, glycosuria, and urine pH modifications were reported in SARS-CoV-2 infection [26,27]. However, as far as we know, the assessment of uEVs in COVID-19 has not yet been investigated. On the other hand, plasma EVs, especially platelet-derived EVs, were reported as biomarkers of the proinflammatory and prothrombotic status associated with COVID-19 [28,29]. Nevertheless, we believe that increased uEV release in the early phase of COVID-19 can indicate cellular stress of renal cells.

Podocyte-derived EVs have been investigated in different scenarios of glomerular injury, with alterations reported in obesity [30], diabetic nephropathy [14,18], renovascular hypertension [31], and systemic lupus erythematosus [32]. In COVID-19, studies have shown that viral particles can be detected on podocytes [33,34] and patients can develop collapsing segmental or focal glomerulosclerosis [35,36]. Despite that, in our study, we observed slightly elevated levels of podocyte uEVs in COVID-19 patients. Thus, one may suggest that the uEVs derived from tubular cells are the major subpopulation of the total uEVs which we identified. In fact, studies reported that SARS-CoV-2 can infect and replicate in the renal tubular epithelium [33,37] and tubular injury is the main renal finding in COVID-19 patients, which is often diagnosed as acute tubulointerstitial nephritis [38]. Thus, further studies are necessary to assess tubule-specific uEVs in COVID-19.

Importantly, we identified that only total uEV levels were able to predict hemodialysis as a clinical outcome in severe/critical COVID-19 patients after the analysis of ROC curves. As mentioned above, patients with severe COVID-19 can develop AKI and require hemodialysis [1,2]; thus, studies have been investigating potential early indicators of kidney injury, which could be potentially considered for a thorough evaluation of these patients. Even though the analysis of proteinuria did not show significant results here, other studies have reported that proteinuria is associated with COVID-19 severity and mortality [26,27,39]. Moreover, in a previous report, we demonstrated that serum biomarkers such as sCr, ferritin, albumin, and CRP were associated with death in a larger cohort [40]. In this study, the lack of significant results for other renal parameters could be due to the small number of patients. Considering this hypothesis, we suggest that total uEVs could predict renal outcomes with superior sensitivity when compared to classical markers of renal dysfunction. This assessment should be considered in future studies for renal risk assessment in other systemic, inflammatory, and infectious diseases, including sepsis.

In the present study, we also observed that urinary levels of cytokines, chemokines, and growth factors were increased in COVID-19 patients, especially in those with severe/critical disease. Lamb et al. (2020) previously demonstrated that COVID-19 patients presented elevated levels of proinflammatory mediators such as IL-6, IL-8, and CXCL-10 in urine [41]. Moreover, Laudanski et al. (2021) demonstrated that high urinary IFN-γ at admission was a good predictor of AKI in COVID-19 patients [42]. Likewise, a proteomic-based study demonstrated that changes in urinary cytokines are associated with AKI development [43]. Taken together, significant alterations in urinary immune mediators may be a reflection of the systemic release, often referred as cytokine storm in COVID-19, and these fluctuations appear to be associated with disease severity.

Recently, our research group demonstrated that circulating levels of several immune mediators are markedly increased in COVID-19 patients who developed AKI independently of death as the final outcome [44]. Here, similar results were found in urine, especially for the alterations in chemokines, such as CCL-2, CCL-3, and CXCL-10, which are known especially for promote monocyte and macrophage migration in addition to being involved in Th1 response and lymphocyte trafficking [45,46]. Moreover, we observed that proinflammatory cytokines involved in lymphocyte and macrophage activation (IL-1β, IL-6, IL-16), Th2 (IL-4), and Th17 (IL-17A) responses were also increased in urine samples of COVID-19 patients [47,48]. On the other hand, urinary levels of IL-9, which can inhibit cytokine production during Th1 response and enhance T cell survival [47,48], were reduced in COVID-19 patients. The same was observed for CXCL-12, a chemokine with a critical role in cell development in different tissues [46].

Lastly, our results demonstrated that total uEVs are significantly correlated with urinary immune mediators in COVID-19 patients. EVs are involved in several physiological and pathological pathways, including the inflammatory process. This is evidenced by previous studies showing associations between cytokines and EVs [49]. In addition, cytokines can be packed into EVs, which can potentialize their effects during acute and chronic inflammation even in distant cells, as recently reviewed by Barnes and Sommerville [50]. On the kidneys, EV signaling can promote tubulointerstitial inflammation via macrophage activation and immune mediators can be released by podocytes and epithelial cells [11].

In COVID-19, besides the association with the proinflammatory state, EVs were proposed as important mediators in renal thrombosis [51]. Indeed, studies have been investigating the effects promoted by plasma EVs from COVID-19 patients. Rausch et al. (2021) reported that increased levels of CD41^+^/phosphatidylserine (PS)^+^ EVs, which can bind to CD8^+^ lymphocytes, were associated with disease severity [52]. Interestingly, Garnier et al. (2022) demonstrated that preincubating EVs isolated from plasma of severe COVID-19 patients with annexin V, and consequently blocking PS exposure, reduces the deleterious EV-mediated effects on endothelial cells [53]. Altogether, we hypothesized that EVs can promote interactions between renal and immune cells, contributing to the establishment and exacerbation of tissue inflammation and this could be reflected as the alterations observed in uEVs and urinary immune mediators. In addition, as previous reports highlighted, understanding EV signaling in SARS-CoV-2 infection could be interesting to develop new therapeutic approaches.

Our study has some limitations. Besides the small cohort, our main obstacle was to obtain urine samples. At the first phase of the pandemic, due to the rapid evolution of severe COVID-19, some patients died within a few hours or days after obtaining the diagnosis. Our work, at the time of conception, was not designed to explore histopathological findings and kidney biopsies in our institution were not largely indicated by assistant physicians for biosafety and epidemiological reasons. Additionally, physicians usually did not require routine urine tests and samples were not available. This also made it difficult to perform the analysis of uEVs and urinary immune mediators on a daily or weekly basis to better understand the kinetics of these biomarkers. Further studies are necessary to better characterize the subpopulations of uEVs in COVID-19 compared to other etiologies of kidney disease.

## 5. Conclusions

Our results show that significant alterations in urinary EVs and immune mediators can be observed early in COVID-19 and are associated with disease severity. Moreover, total uEVs and immune mediators are significantly correlated with one other. This suggests that, besides the direct effects of SARS-CoV-2 on the kidneys, EV release could also be promoted by immune activation as a consequence of the multisystemic inflammation. Thus, alterations in these biomarkers could identify patients at higher risk of developing renal dysfunction.

## Figures and Tables

**Table 1:** Demographic and clinical characteristics of COVID-19 hospitalized patients and healthy controls.

Parameters	HC n = 7	COVID-19		*p*-Value
		Mild/Moderate (n = 24)	Severe/Critical (n = 12)	
Age, mean ± SD	42.3 ± 8.2	53.5 ± 20.5	62.6 ± 13.1	0.06
Female gender, n (%)	5 (71.4)	15 (62.5)	5 (41.7)	0.3
Comorbidities, n (%)				
Hypertension	-	14 (58.3)	8 (66.7)	0.6
Coronary artery disease	-	2 (8.3)	1 (8.3)	0.9
CKD stage 2/3 *	-	1 (4.2)	1 (8.3)	1.0
Diabetes	-	3 (12.5)	5 (41.7)	**0.04**
Oncohematological disease	-	4 (16.7)	2 (16.7)	0.6
Laboratory tests, mean ± SD				
sCr (mg/dL)	-	1.1 ± 0.7	2.3 ± 2.6	0.10
Urea (mg/dL)	-	39.4 ± 22.6	68.2 ± 52.2	**0.03**
uPtn/uCr (mg/g)	131.4 ± 126.4	524.4 ± 641.1	2222 ± 2640	**0.001 ^b^**
ACR (mg/g)	3.9 ± 1.6	62.8 ± 119.5	485.8 ± 710.9	**<0.0001 ^a,b,c^**
Outcomes, n (%)				
ICU admission	-	4 (16.7)	7 (58.3)	**0.01**
Hemodialysis	-	-	4 (33.3)	**0.002**
Death	-	1 (4.1)	5 (41.7)	**0.004**

Data is presented as n (%) or mean ± standard deviation. *p*-values were calculated using Chi-square, Mann–Whitney test or Kruskal–Wallis with Dunn’s post-test (^a^, HC vs. Mild/Moderate; ^b^, HC vs. Severe/Critical; ^c^, Mild/Moderate vs. Severe/Critical) and were considered statistically significant when *p* < 0.05 (in bold). ACR = albumin-to-creatinine ratio; * CKD = chronic kidney disease (glomerular filtration rate 60–45 mL/min/1.73 m^2^); Cr = creatinine; HC = healthy control; ICU = intensive care unit; Ptn = protein; s = serum; u = urinary.

**Table 2:** Correlations between total urinary EVs and immune mediators.

Parameters	*p*-Value	Parameters	*p*-Value
TNF-α	r = 0.87 *p* < 0.0001	CCL-3	r = 0.65 *p* < 0.0001
IL-1α	r = 0.88 *p* = <0.0001	CCL-4	r = 0.68 *p* < 0.0001
IL-1β	r = 0.85 *p* < 0.0001	CXCL-9	r = 0.53 *p* = 0.001
IL-4	r = 0.62 *p* = 0.004	TRAIL	r = 0.87 *p* < 0.0001
IL-8	r = 0.70 *p* = <0.001	HGF	r = 0.86 *p* < 0.0001
IL-9	r = 0.71 *p* < 0.0001	LIF	r = 0.39 *p* = 0.02
IL-16	r = 0.85 *p* < 0.0001	FGF-B	r = 0.57 *p* < 0.0001
IL-17A	r = 0.80 *p* < 0.0001	PDGF-bb	r = 0.67 *p* < 0.0001

r = Pearson’s coefficient.

## Data Availability

Not applicable.

## Supplementary Materials

The following supporting information can be downloaded at: https://www.mdpi.com/article/10.3390/diagnostics12112600/s1, Table S1: Linear regression models for analyzing independent variable’s influence on total uEV levels; Table S2: Urinary levels of immune mediators in COVID-19 hospitalized patients and healthy controls. Supplementary Data S1.

## Abbreviations

ACR	albumin-to-creatinine ratio
CAD	coronary artery disease
CCL	C-C chemokine ligand
CKD	chronic kidney disease
Cr	creatinine
CXCL	C-X-C ligand
EV	extracellular vesicles
FGFb	basic fibroblast growth factor
GM-CSF	granulocyte-macrophage colony stimulating factor
HGF	hepatocyte growth factor
ICU	intensive care unit
IFN	interferon
IL	interleukin
LIF	leukemia inhibitory factor
MCP-1	monocyte chemoattractant protein 1
MIF	migration inhibitory factor
PDGF-BB	platelet-derived growth factor BB
Ptn	protein
s	serum
SCF	Stem cell factor
SCGF-beta	Stem cell growth factor
TNF alpha	tumor necrosis factor- alpha
u	urinary
