# Supplementary material for: Exploring Urinary Extracellular Vesicles and Immune Mediators as Biomarkers of Kidney Injury in COVID-19 Hospitalized Patients
Source: Diagnostics (Basel). 2022 Oct 27;12(11):2600. doi: 10.3390/diagnostics12112600 (PMC9689919; doi:10.3390/diagnostics12112600)
Supplement: Supplementary file 1 [file diagnostics-12-02600-s001.zip › diagnostics-1910395-supplementary-tables.pdf]

Table S1. Linear regression models for analyzing independent variable's influence on total uEV levels

Models	Regression coefficients	Std Error	*P*-value
Age	23.29	68.15	0.734
Diabetes	472.65	3124.10	0.881
Hypertension	-1563.98	2644.72	0.558

Table S2. Urinary levels of immune mediators in COVID-19 hospitalized patients and healthy controls

Parameters (pg/μL)	HC n= 7	Mild/Moderate (n=24)	Severe/Critical (n=12)	*P*-value
**Pro-inflammatory cytokines**				
IL-1α	1.1±0.9	2.9±3.7	3.3±3.5	0.5
IL-1β	0.77±0.3	0.94±0.6	1.9±1.2	**0.02**[b, c]
IL-4	0.26±0.2	0.21±0.15	1.04±1.02	**0.0003**[b, c]
IL-5	10.0±4.6	9.7±5.2	8.0±4.6	0.51
IL-6	0.33±0.31	2.5±6.0	177.6±456.2	**0.0005**[b, c]
IL-7	10.3±6.3	8.0±6.0	14.6±4.9	**0.02**[c]
IL-8	3.6±2.7	60.0±158.7	33.4±50.9	0.09
IL-9	5.6±2.5	2.8±1.7	2.7±1.8	**0.02**[a, b]
IL-13	0.17±0.06	0.27±0.48	0.28±0.14	**0.02**[c]
IL-16	0.70±0.27	3.5±6.8	42.5±58.6	**0.003**[b, c]
IL-17A	2.3±0.7	2.2±1.7	7.0 ±8.8	**0.04**[c]
IL-18	4.2±2.0	7.9±7.4	11.7±5.0	**0.003**[b]
TNF-α	8.3±0.8	14.0±13.5	13.8±7.6	0.3
IFN-γ	13.4±3.3	23.3±25.7	67.4±52.8	0.05
LIF	7.2±3.6	9.6±6.7	53.2±50.3	**0.03** [b]
MIF	53.3±18.3	190.1±387.4	555.2±589	**0.01**[c]
**Anti-inflammatory cytokines**				
IL-1Ra	2856±2724	19806±14112	25212±11483	**0.02**[a,b]
IL-2Ra	57.3±15.5	86.3±136.5	73.4±54.0	0.9
**Chemokines**				
CCL-2	94.9±63.7	265.2±607	47074 ±30129	**0.0001**[b,c]
CCL-3	0.15±0.1	1.1±2.1	2.8 ±3.9	**0.03**[b]
CCL-4	3.3±2.1	1.3±1.7	2.5±2.8	**0.04** [a]
CCL-5	477.9±825	9.1±12.8	15.9±14.7	**0.03** [a]
CCL-11	14.9±4.0	11.7±15.6	134.6±175.1	**0.02**[c]
CXCL-9	60.2±44.5	236.8±842.4	215.2±424.6	0.06
CXCL-10	3542±3383	5215±8783	92838±104934	**0.002**[b,c]
CXCL-12	196408±320252	12485±46187	12645±16856	**0.04**[a]

Supplementary Table S2. (cont)

**Parameters (pg/µL)**	**HC n= 7**	**Mild/Moderate (n=24)**	**Severe/Critical (n=12)**	***P*-value**
**Growth factors and others**				
PDGF-BB	6.7±1.9	5.2±2.1	5.7±3.5	0.25
FGF-B	8.7±1.6	9.6±3.3	15.7±9.3	**0.008**[b,c]
M-CSF	159.4±128.1	230.3±224.5	444.1±259.6	**0.01**[b,c]
SCF	10.4±.6.9	39.8±69.8	104.5±158.8	0.06
SCGF-β	56.3±58.8	112.5±89.0	82.7±88.6	0.23
HGF	21.6±13.4	26.5±23.5	64.5±51.9	0.15
TRAIL	1.1±0.8	1.3±1.1	2.1±2.3	0.91
CTAcK	7.3±2.7	5.6±6.0	15.44±13.5	**0.01**[a,c]

Data is presented as n (%) or mean ± standard deviation. P-values were calculated using ANOVA test and post-test Turkey or Kruskal Wallis test post-test Dunn' (a, HC *vs* Mild/Moderate; b, HC *vs* Severe/Critical; c, Mild/Moderate *vs* Severe/Critical) and were considered statistically significant when $p < 0.05$ (in bold).
